# Situational analysis on the recovery of patients who have undergone major abdominal surgery

**DOI:** 10.1002/nop2.612

**Published:** 2020-09-07

**Authors:** Warawan Udomkhwamsuk, Nuttamon Vuttanon, Sirikan Limpakan

**Affiliations:** ^1^ Faculty of Nursing Chiang Mai University Chiang Mai Thailand; ^2^ Faculty of Medicine Chiang Mai University Chiang Mai Thailand

**Keywords:** major abdominal surgery, postoperative patients, recovery, situational analysis

## Abstract

**Aim:**

To analyse the recovery situation of patients who underwent abdominal surgery.

**Design:**

A descriptive study.

**Method:**

This study was conducted among 50 participants: 15 postoperative patients, 16 caregivers, 2 surgeons and 17 nurses in a tertiary hospital in Thailand. The state of patients’ recovery after undergoing major abdominal surgery was analysed using Donabedian's approach.

**Results:**

The findings showed that hospitals and some organizations do not have a clear policy about clinical care to help patients recover after undergoing major surgery or to prevent the risk of complications following major abdominal surgery. In addition, there were no clinical practice guidelines in use in each ward. Each ward should have a set of guidelines and procedures for assisting with patient recovery. The procedures should be based on nursing care. It is necessary to have a coordinated multidisciplinary care guideline to use with other health professionals to promote the recovery of patients.

## 1 INTRODUCTION

Abdominal surgery is any operation on the intra‐abdominal organs to repair accidental injury, peritonitis, abdominal abscess and hepatic abscess (Kapoor et al., 2017). Most postoperative patients have a lot of body tension, little movement, and take shallow breaths due to the pain and fear of wound separation. Inactivity for long periods of time causes body fatigue and coupled with fatigue from illness or dysfunction in other bodily systems, and this fatigue will eventually cause complications. It was also found that the duration of hospital stays for patients undergoing abdominal surgery is longer than for other procedures, with an average hospital stay of 13–36 days (Howes et al., 2015). A long stay can adversely impact the patient financially. Patients normally suffer following abdominal surgery, and this suffering affects the patient physically, mentally, emotionally and socially (Buttenschoen et al., 2009).

The recovery period after abdominal surgery falls into three phases (Boyer & Royse, 2016). The first phase, or early postoperative recovery phase, includes the first 7 days following surgery. Patients are able to move their body and can breathe normally, and their vital signs begin to stabilize. The next phase is called the intermediate phase of postoperative recovery. This period encompasses 7–60 days after surgery. The patient's body begins to stabilize, and the patient is able to engage in more daily activities. During this period, patients may have various complications such as pain, fever, deep vein thrombosis and inflammation, wound infection, stress and anxiety. Finally, the late recovery phase is counted from 6 weeks to 3 months after surgery. Patient recovery is faster, and more daily activities can be performed than previously.

The biggest impact abdominal surgery has on patients is the inability to perform daily activities and needing help from others. In addition, patients experience a feeling of discomfort due to severe pain from the surgical wound and muscle contraction for two or three days after surgery. A postoperative complication resulting from the severe pain is the patient's refusal to ambulate. The refusal to ambulate can result in other side effects such as lung infection, bloating, nausea and vomiting (Gan, 2017). In addition, these postoperative patients also affect the hospital's resources. Beds taken by postoperative abdominal patients could have been used to accommodate the needs of other patients. This also affects patients as a longer hospital stay means increased expenses for the patient. Patient care after abdominal surgery differs depending on the type of operation. Different types of care result in different quality of health service (Kibler et al., 2012). Promoting postsurgical recovery with a quick return to normal functioning is important. Physical recovery after surgery differs depending on various factors such as age, congenital disease, the psychological and social condition of patients, surgery type, wound size, pain type and duration of surgery. If vital signs and circulation during surgery are not very stable, these factors can affect recovery. (Trang, Thosingha, & Chanruangvanich, 2017). Therefore, effective postoperative care should not only focus on reducing complications but also help to reduce length of hospitalization. A systematic analysis of the problems related to postoperative care for abdominal surgery was conducted to better understand structure, process and results. The research question of this study was what the recovery situation of patients with abdominal surgery is provided. The objective of this study was to analyse the recovery situation of patients with abdominal surgery.

## 2 METHODS

A descriptive research design was used, and postoperative patients, caregivers and health professionals in the surgical ward were targeted. Participants came from a tertiary hospital in Chiang Mai province between August 2017 and February 2019. A convenience sampling technique was used to recruit participants. The study used Donabedian's conceptual framework (Donabedian, 1988) which explores the linkage between structural, process and outcomes with the service quality assessment.

This study used both descriptive research and qualitative research, and the following research instruments were used:

1. Sociodemographic questionnaire to collect information on gender, age, nationality, religion, education level, career, status and lifestyle.
2. Guidelines for group discussion, in‐depth interviews and participatory observation, developed by the researchers.
3. Record review form for noting information from abdominal surgery records in the surgical department.

### 2.1 Data collection

Record review was used to collect information on patient history and treatment, and recovery information following abdominal surgery. The questionnaire was admnistered to patients after they gave consent for study. This was followed by the qualitative component, three group discussions were conducted, one each for nurses, patients undergoing abdominal surgery, and family members and caregivers of these patients. Each group had between 6–8 participants. In‐depth interviews were conducted with the surgeons, nurses, patients and caregivers. All discussions and in‐depth interviews were audio recorded.

### 2.2 Rigor and trustworthiness

Strategies to enhance trustworthiness were applied to ensure the rigor of this study. Credibility was strengthened multiple methods of data collection. Methodological triangulation was used to compare a variety of data sources and methods in order to confirm the accuracy of the findings. Observations of participant behaviour, facial expressions and the surrounding environment were recorded in the field notes at the end of each meeting. Participatory activities and in‐depth interviews were used to confirm the accuracy of findings. Member checking is one strategy to confirm credibility, and some participants were asked to validate and give feedback on the data they provided. Furthermore, the findings were shared with the research team to verify accurate interpretation. A clear audit trail showed all findings were derived directly from the data, ensuring confirmability.

### 2.3 Data analysis

In this research, data obtained from the research process included both qualitative and quantitative data. The data analysis was divided into two parts as follows: (a) Quantitative data were calculated using frequency and percentage. (b) Qualitative data were analysed iteratively using thematic analysis (Miles & Huberman, 1994). The transcripts in each group were read, and categories were reviewed several times in order to ensure that concepts pertaining to the same phenomena were placed in the appropriate category. The themes and the content of the data throughout the data collection and process analysis were identified by the primary author and subsequently verified by two co‐authors for coding consistency, emergence of main themes and extraction of statements to support the themes. Coding, themes and key findings were discussed by the co‐authors until a consensus was reached.

## 3 RESULTS

In Table 1, the health professional sample was comprised of 19 hospital employees, 2 physicians and 17 nurses, all female and the majority of whom had a bachelor degree (61.11%). More than a third were between 30–39 years old (38.89%) and had between 6–10 years of experience in the surgical ward. The patient and caregiver sample included a total of 31 participants, 16 caregivers and 15 patients, comprised of 23 women (71.9%), 13 of whom (41.9%) had a primary school education. The majority of this group (67.7%) had an average monthly income of <10,000 baht. Ten worked in gardening, and nine worked as office staff, accounting for 32.2% and 29.0% of the sample, respectively.

**Table 1:** Demographic data represent number, and percentage of the population classified by sample group

Population characteristics	Numbers	Per cent
Sex		
Health professional		
Male	0	0
Female	19	100
Caregivers and patients		
Male	8	28.1
Female	23	71.9
Age groups (years)		
Health professional (*N* = 19)		
20–29	5	27.78
30–39	7	38.89
40–49	4	22.22
>50	2	11.11
Care givers and patients (*N* = 31)		
20–29	4	12.90
30–39	4	12.90
40–49	6	13.36
>50	17	22.58
Education level		
Health professional (*N* = 19)		
Bachelor degree	12	61.11
Master degree	7	38.89
Care givers and patients (*N* = 31)		
Uneducated	5	16.1
Primary school	13	41.9
Secondary school	7	22.6
Bachelor degree	6	19.4
Care givers and patient's occupations (*N* = 31)		
Not working	6	19.4
Trade	3	9.7
Government service	3	9.7
Gardening	10	32.2
Employee	9	29.0
Care givers and patient's outcomes (Baht) (*N* = 31)		
<10,000	21	67.7
10,001–20,000	7	22.6
20,001–30,000	1	3.2
30,001–40,000	1	3.2
40,001–50,000	1	3.2

### 3.1 Part 1 Organizational structure

Surgical and postsurgical care was divided by medical team member type and specialization by system. The physician team provided care according to their specialization, for example liver and bile duct system, upper gastrointestinal and lower gastrointestinal system. The physician team consisted of medical professors, residents or interns with 1–3 years of training in the area specialization they were working, and 6th year medical students assigned to 1–3 month rotations for each system. The nursing team was made up of registered nurses, practical nurses and nursing assistants who were responsible for caring for patients based on two overarching systems, namely the chest and gastrointestinal system. The goal of the nursing team was to provide care according to professional standards to allow patients to recover quickly and without disability after major abdominal surgery. Personnel staffing for postoperative patients varied by time of day. In the morning, staffing included four registered nurses, two practical nurses and two assistants. The afternoon and night shift included two registered nurses and one practical nurse per patient. During the evening shift, the registered nurse to patient ratio was 1:4, markedly lower than the daytime shift. In addition to the nursing team, physical therapists provided consultations for recovery after surgery. Physical therapy consultations were provided by patient request or for patients with complications.
Policy. The hospital did not have a clear policy about the use of clinical practice in helping patients recover after undergoing major surgery. In addition, there were no clinical practice guidelines for each ward. Each ward should have a set of guidelines and procedures in assisting the recovery of patients. The procedure is based on the basic surgery nursing care support.
Organization management. This tertiary hospital had 20–22 beds in each ward. This hospital was part of a medical institution based at a university. Surgical patient care was divided between wards for the intensive care unit surgery and general surgery. These wards were further divided by gender, and each gender was provided care in different wards by based on body systems. The general surgical ward was divided into cardiovascular and coronary surgery, thoracic and pulmonary surgery, gastrointestinal surgery, and urology surgery and plastic surgery. While the intesive care unit surgical ward was also divided by body systems, it had fewer divisions than the general surgical ward.

Most patients who underwent major abdonimal surgery were admitted to the ward treating biliary and digestive tract. Most patients underwent surgery due to haemorrhages, infections and inflammation of the abdominal organs, or due to tumours or cancers in the abdominal organs. The biliary and digestive tract ward had eight beds, and most of the patients in this ward were treated for gastrointestinal disorders. The hospital also accepted patients from the outpatient or emergency departments of nearby hospitals. Consultations were done for patients with gastrointestinal disorders and those requiring surgery from different departments in the hospital such as the internal medicine department or paediatric department. When the doctor determined that the patient had to undergo surgery, there was coordination to reserve a spot in the operating room queue and to prepare the patient.

### 3.2 Part 2 Establishing a recovery system for patients undergoing major abdominal surgery

#### 3.2.1 Direct patient care

After receiving patients for care, the physician team assessed the patient's condition in every system for diagnosis and to develop an appropriate treatment plan. This assessment included evaluation of objective and subjective data, co‐morbid conditions, malnutrition, physical examination and interpretation, routine laboratory examination and special examinations. The physician team considered all components in clinical decisions and care practices for patients. The healthcare team made preparations beginning with the stage before the surgery. For example, they assessed the risks that may occur during the surgery and the postoperative period, they provided health education to the patient before surgery, and they consideration the different surgical methods. This entire process helped patients to recover quickly after surgery and without disabilities. The physician team also coordinated with the anaesthetist team to assess the surgical risks such as blood transfusions, care in receiving cardiovascular stimulants and surgery duration. During the postoperative period, patient symptoms were monitored in the intensive care unit and the sub‐intensive care unit. Following surgery, the medical team visited patients twice a day to monitor patient symptoms. The level of counselling from the medical team began with 6th year medical students, interns, 1st year residents, 2nd year residents and 3rd year residents, respectively. During the examination, the following information was reviewed: symptoms in the patient record, a physical examination information, laboratory results, and information relating to traumatic surgical wound pain and secretions from various vents.

#### 3.2.2 Integration of empirical evidence into practice

Empircal evidence is gained from relevant research, both medical and nursing, and from previous experience in patient treatment. Using the research evidence combined with clinical expertise and knowledge of pathophysiology in managing patient problems and treatment decisions is important in achieving good patient outcomes.

This study showed that evidence‐based care provided by nurses was not fully up to date. Some areas of care used the most current evidence, whereas other areas relied on old standards. The best example of evidence‐based care wass in the creation of a specific team to provide care for the hepatectomy clinical care pathway. Because nurses were assigned to work in different clinical areas and were responsible for several diseases, compliance with guidelines was not consistent.

Aside from hepatectomy, there were no clear guidelines for patient care plans for other major abdominal surgeries. Also absent were clear clinical practice guidelines to prevent the risks and complications following major surgery such as guidelines for the prevention of wound infections, guidelines for patient recovery after major surgery and guidelines for referrals following discharge from the hospital.

#### 3.2.3 Training staff to develop knowledge about helping patients to recover

Periodic group meetings were held between the head nurse and the care team to improve nursing skills, nursing quality and problem solving skills in patient care. However, holding regular meetings was unsuccessful due to time constraints. Intervention and supervision between supervisors and nurses were not always possible. In addition, there was a lack of coordination and few treatment team meetings between doctors and nurses to solve problems in patient cases. As a result, the recovery stage after major abdominal surgery was comprised of medical care only.

In the treatment department of the medical team, there were regular treatment team meetings to discuss patient problems and to decide on the course of treatment based on guidance from the team leader or a majority team vote. These meetings were held twice a day during the morning and evening visits. For complex cases involving more challenging diseases or pathologies, the hospital did not seek consultation with medical professors. They also did not use a multidisciplinary team meetings of doctors, nurses, physical therapists and nutritionists to plan patient recovery after surgery.

Both doctors and nurses provided patient education to prepare them on what to expect before and after surgery. Doctors provided information on the specifics of surgery and advised them not to drink water or eat food before surgery and performed a physical examination with the anaesthesiologist. Nurses provided education on self‐care such as breathing instruction, effective coughing and getting out of bed quickly. Patients were given medication to relieve anxiety before surgery. Although the healthcare team did a good job of teaching and preparing patients on what to expect before and after surgery, there was no formal evaluation of patient knowledge about postoperative care.

#### 3.2.4 Management of medical tools and equipment

The hospital provided medical tools and equipment for the surgery and adequate medical supplies for patient care. Sterile equipment was provided by the central distribution unit under the management of the ward housekeeper. Various medical supplies that were used before and after surgery, such as analgesic drugs and antimicrobial drugs, were all in sufficient amount for treatment. It is important to note that an incentive spirometer is an important piece of equipment to assist in patient recovery. This was not provided by the hospital, and its purchase was the sole responsibility of the patients and their relatives.

### 3.3 Part 3 The outcome of the rehabilitation service system for patients undergoing major abdominal surgery

While some patients were satisfied with their care, most commented that medical personnel, both physicians and nurses, were overburdened and that recovery care for postoperative patients was not as effective as it could have been. Some patients experienced complications after surgery such as bleeding. In addition, there was no clear record in the patient charts of complications experienced by each patient or the result of care for these complications.

Data from the in‐depth interviews and group discussions found that recovery in patients after major abdominal surgery could be summarized in three topics as demonstrated by the following statements:

#### 3.3.1 Lack of clear guidelines for postoperative care for patients

Both doctors and nurses lack clear clinical practice guidelines. (Nurse)

There is no set course based on the patient's condition. (Doctor)

There are no clear guidelines. (Nurse)

Mostly it is the knowledge that has brought us. (Nurse)

#### 3.3.2 Patients do not cooperate in recovery

Patients do not cooperate in recovery because of wound pain. They are not interested in advice from the healthcare team, resulting in an inability to recover effectively. Because of patient cultural diversity, some information is not effectively communicated because patients do not always speak Thai.

Mostly, patients have severe pain; they are unable to ambulate. (Nurse)

Some patients are from hill tribes and are unable to communicate with health care professionals and will have to wait for their caregivers to help them communicate. (Nurse)

#### 3.3.3 There is a need for multidisciplinary coordination to help in recovery

In order to help the patients recover after surgery, there should be coordination among multidisciplinary teams in promoting and caring for patients for effective recovery.

The system should have multidisciplinary teams to help in the recovery. Doctors alone with a physiotherapist cannot solve this problem. (Doctor)

The hospital should have a multidisciplinary team to take care of postop patients. (Nurse)

## 4 DISCUSSION

This study examined the structure that a health facility had in place to help patients recover from major abdominal surgery. Most patients were admitted to the general surgical ward in the public hospital, which was not only responsible for the care of patients with abnormalities in the gastrointestinal and hepatobiliary system but also for patients who have undergone surgery in other systems such as pulmonary, cardiovascular and renal. Accepting patients into ward care mainly came from the outpatient department or the emergency department with a few patients who have moved from other wards such as the intensive care unit and medical ward. Care in the ward was based on pathophysiology related to the expertise of the surgical and medical treatment team, including residents and interns in specialized orthopaedic training supervised by a medical professor. In the wards, care from the nursing team was also based on body system and the nursing care ratios were suitable. Donabedian (2005) stated that process measures reflect the systems and processes work to deliver the desired outcome. For example, the length of time a patient waits for a senior clinical review and the length of waiting time for operational periods, whether a patient receives certain standards of care or not, and whether healthcare professions record incidents and act on the findings. All of these affect outcome measures.

For patients with problems during recovery, a physical therapist will be sent. The arrangement between the nurse and a case manager in having a multidisciplinary approach in coordinating patient care to recover helps promote an efficient and faster postoperative recovery (Kibler et al., 2012).

In establishing a recovery system for patients after major abdominal surgery, the medical team has prepared for the patients before and after surgery. There is a patient preparation plan for surgery, and the required surgical equipment is available. The medical team determined the pathology of the disease and the associated diseases in each patient. In addition, a team of anaesthetists coordinates to assess risk during surgery and the stage of surgery. During the postoperative period, patients' symptoms are monitored in the intensive care unit. After the operation, the patient receives a series of visits to assess the recovery progress and clinical symptoms. Patient recovery and complication such as pain, postoperative cognitive dysfunction differs from depending on the pathology of each patient, surgical procedure and anaesthesia (Czyż‐Szypenbejl, 2019). Nurses should try to detect postoperative complications early using instruments that have been tested such as the Clavien–Dindo classification system (Dindo, Dermartines, & Clavien, 2004) and notify the physician of any abnormal conditions. In terms of medical care, the team of doctors and nurses apply evidence‐based practice, integrating the information from relevant research and their personal experiences. Most treatments provide treatment plans and assessment of patients' recovery based on the pathology and physical condition of each patient. What is lacking are clear clinical practice and comprehensive guidelines to prevent complications. Comprehensive guidelines have been effective in speeding up recovery after a major surgery. Nurse should use evidence‐based practice (EBP) by applying in daily practice acute pain management during the postoperative period in older patients (Medrzycka‐Dabrowska, Dabrowski, Gutysz‐W, & Basinski, 2016). In Thailand, the nurse administers an analgesic on demand with a doctor's order prescribing and adjusting the analgesic dose are the responsibilities of a physician in all cases. Therefore, it is difficult to follow evidence‐based practice guideline from other countries.

Additionally, the lack of coordination meetings between the multidisciplinary team hinders staff's performance in helping patients recover. While this hospital had elements of pre‐operative patient education in place, there was no formal pre‐operative preparation programme. Sritharn (2017) found that abdominal surgery patients receiving a pre‐operative preparation programme had a better postoperative recovery than those who only received normal nursing care. Tool preparation and medical equipment including the pharmaceuticals that are relevant enough to take care of patients are important.

Having the equipment ready is only one part in helping patients to have an effective recovery after surgery and to shorten the length of their hospital stay. A good recovery relies on patient care practices that address the patient's physical, mental, emotional, social and routine functions. The patient should be physically comfortable with helping themselves in their daily activities. Patients should have range of motion, so they can perform various activities independently. Patients without complications who experience little pain have a happy emotional and mental state and are calm, without depression, are able to have proper interaction with other people and society (Allvin, Berg, Idvall, & Nilsson, 2007). Emergency operations and providing treatment in a way that ensures a quick recovery that is without complications is not possible without clear practice guidelines for multidisciplinary patient care. Therefore, multidisciplinary teamwork must be strengthened to ensure a readiness to make changes in surgical patient outcomes, especially in the reduction of hospital stay, rate of complications, early recovery and reduction of economic burdens (Taurchini, Naja, & Tancredi, 2018), other communities. Since this study was conducted in tertiary hospital in Chiang Mai Province, it was unable to represent the larger population of postoperative service in Thailand. This study was conducted in a specific Thai tertiary hospital, and the process and steps of postoperative service may not be generalized to other settings or contexts. Additionally, as this study includes only gastrointestinal and biliary surgery patients, it did not provide data about urology and gynaecology surgeries.

## 5 CONCLUSION

This research finding provide the surgery care system in tertiary hospitals in Thailand about clinical care to help patients recover after undergoing major surgery or to prevent the risk of complications following major abdominal surgery. It is necessary to have a coordinated multidisciplinary care guideline to use with other health professionals to promote the recovery of patients after surgery. There should be coordination between multidisciplinary teams in promoting and caring for patients to allow for effective recovery.

## CONFLICT OF INTEREST

The authors declared no potential conflict of interest with the research, authorship and publication of this article.

## ETHICAL APPROVAL

Research Ethics Committee approval was obtained from the Chiang Mai University Faculty of Nursing Research Ethics Committee and the Faculty of Medicine Research Ethics Committee. Following IRB approval, permission to conduct the study was also granted from the hospital administrator. All participants were informed in advance about the purpose and research process of this study. They were informed that participation in this study was voluntary. Participants were assured that none of the information gained in the study could affect their daily lives or job. All data were treated as group information with no personal identifiers. Individuals who agreed to participate were asked to sign a consent form. Participants were told that they had the right to refuse to answer any of the questions posed at any time during the interview and the right to stop the recording during the interview at any time they chose. All of the written data, including reflection and field notes, were destroyed, and all audio recordings were erased after the study was completed. A token of appreciation was given to each participant at the end of the study.

## Data Availability

The data that support the findings of this study are openly available in data at https://o365cmumy.sharepoint.com/:f:/g/personal/warawan_u_cmu_ac_th/EuZ3D2kHPbpFn7LVKSb6S6kBckq9uWYqlz‐tHOBWrCuuWA?e=YvXzAl
